# Antipsychotics-Loaded Nanometric Emulsions for Brain Delivery

**DOI:** 10.3390/pharmaceutics14102174

**Published:** 2022-10-12

**Authors:** Patrícia C. Pires, Ana Cláudia Paiva-Santos, Francisco Veiga

**Affiliations:** 1 Department of Pharmaceutical Technology, Faculty of Pharmacy of the University of Coimbra, University of Coimbra, 3000-548 Coimbra, Portugal; 2 REQUIMTE/LAQV, Group of Pharmaceutical Technology, Faculty of Pharmacy of the University of Coimbra, University of Coimbra, 3000-548 Coimbra, Portugal; 3 Health Sciences Research Centre (CICS-UBI), University of Beira Interior, Av. Infante D. Henrique, 6200-506 Covilhã, Portugal

**Keywords:** antipsychotic, brain delivery, microemulsion, nanoemulsion, nanometric emulsion, schizophrenia

## Abstract

Antipsychotic drugs have numerous disabling side effects, and many are lipophilic, making them hard to formulate at high strength. Incorporating them into nanometric emulsions can increase their solubility, protect them from degradation, and increase their brain delivery, being a promising strategy to overcome the current treatment gap. A thorough review was performed to assess the true potential of these formulations for antipsychotic drugs brain delivery. Intranasal administration was preferred when compared to oral or intravenous administration, since it allowed for direct brain drug transport and reduced systemic drug distribution, having increased efficacy and safety. Moreover, the developed systems increased antipsychotic drug solubility up to 4796 times (when compared to water), which is quite substantial. In the in vivo experiments, nanometric emulsions performed better than drug solutions or suspensions, leading to improved brain drug targeting, mainly due to these formulation’s excipients (surfactants and cosolvents) permeation enhancing capability, added to a small droplet size, which leaves a large surface area available for drug absorption to occur. Thus, even if it is difficult to conclude on which formulation composition leads to a best performance (high number of variables), overall nanometric emulsions have proven to be promising strategies to improve brain bioavailability of antipsychotic drugs.

## 1. Schizophrenia: Pathophysiology, Current Therapy and Treatment Gap

**Figure 1:**
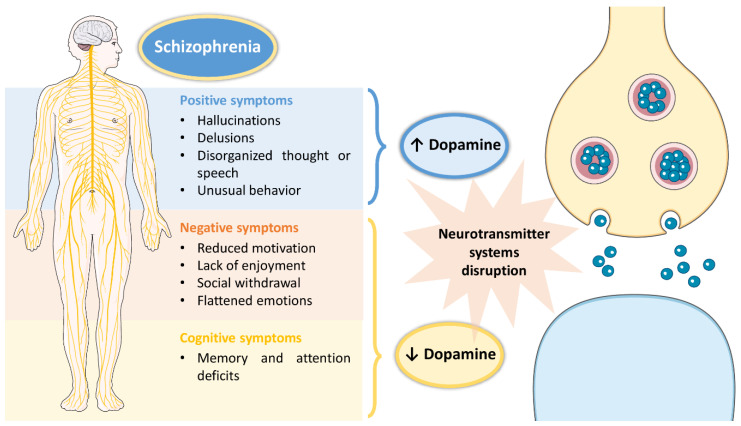
Pathophysiology of schizophrenia, including associated symptoms and pathological mechanisms at the cellular level.

Schizophrenia is a high incidence psychiatric disorder, having been recently estimated to affect 21 million people worldwide [1]. Its onset usually occurs in early adulthood and has a high impact on a patients’ quality of life, since it is characterized by a set of symptoms that not only affect the individual’s day-to-day functioning, but also their surroundings [1,2]. These symptoms include: hallucinations, delusions, disorganized thought or speech, and unusual behavior as positive symptoms; reduced motivation, lack of enjoyment, social withdrawal and flattened emotions as negative symptoms; and memory and attention deficits as cognitive symptoms [2,3]. Although the exact pathophysiology of schizophrenia remains unclear, disruptions in several neurotransmitter systems (serotonergic, cholinergic, glutamatergic, GABAergic, dopaminergic) have been associated with the disease [1,2,3]. However, the most affected neurotransmitter system in schizophrenic patients seems to be the dopaminergic system, with the existence of a dysfunction in dopamine production, which either increases (associated with positive symptoms) or decreases (associated with negative and cognitive symptoms) (summary of the pathophysiology of schizophrenia in Figure 1).

Therefore, the chosen pharmacological treatment in a clinical context are usually antipsychotic drugs that mostly target dopamine receptors. Dozens of different drugs are available in the market and, hence, the treatment has to be tailored to the individual [1,2,4]. Antipsychotics can also be used to treat other schizoaffective or delusional disorders (alone or in combination with other drugs), such as acute mania, major depressive disorder with psychotic features, delusional disorder, severe agitation, Tourette syndrome, borderline personality disorder, dementia and delirium [5]. Nevertheless, in general, antipsychotic drugs lead to a large number of disabling side effects (metabolic, cardiovascular, neurologic), and many times result in relapse and treatment resistance. These limitations many times lead to treatment discontinuation [1,3]. Consequently, safer and more effective treatment options are urgently required. Yet, since many of these molecules have limiting aqueous solubility issues, due to being highly lipophilic, they are very hard to formulate, in technological terms.

## 2. Potential of Nanosystems for Brain Drug Delivery

**Figure 2:**
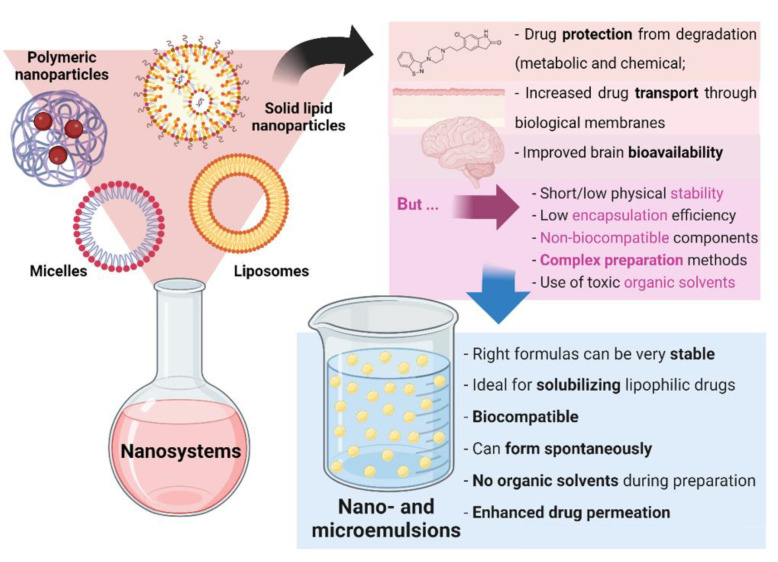
General nanosystem types and their characteristics, as well as the advantages of nano and microemulsions.

Nanosystems, whose colloidal structure has a mean diameter of less than 500 nm, are drug delivery systems that have been showing a high number of advantages for biomedical applications. Aside from increasing drugs’ solubilization and protecting them from degradation (both metabolic and chemical), these structures increase drug transport through biological membranes and, ultimately, promote improved brain bioavailability. Since the very low permeability of the blood-brain barrier blocks the entrance of the grand majority of molecules (especially hydrophilic and/or high molecular weight compounds), this performance is essential for diseases with a brain etiology, in which brain targeting is required, being thereby capable of decreasing the systemic bioavailability, prompting to increased safety due to less systemic side effects, while increasing the therapeutic efficacy [6,7,8,9,10]. There are several types of nanosystems, with the main categories being: polymeric nanosystems (polymeric nanoparticles, polymeric micelles), lipid nanoparticles (solid lipid nanoparticles, nanostructured lipid carriers), liposomes and related nanosystems (transfersomes, cubosomes, ethosomes), and nanometric emulsions [11]. Nevertheless, most of these systems have been reported to have short/low physical stability, low encapsulation efficiency, and non-biocompatible components, while requiring complex preparation methods, that are time- and resource-consuming, and that frequently use toxic organic solvents [12,13,14]. Yet, nanometric emulsions, which consist of colloidal liquid-in-liquid dispersions, can form spontaneously, evidencing the great advantage of having straightforward methods of preparation, that are not quite common. They can also be prepared by high-energy methods (such as by using high-pressure homogenizers, ultrasonication, or extrusion through a small pore synthetic membrane), but spontaneous emulsification is possible when its components are in the right proportions. Moreover, their preparation does not require the use of toxic organic solvents. They are made of a water phase, oils, surfactants, and cosurfactants or cosolvents, and can be classified according to the nature of their internal and external phases, being divided into oil-in-water (O/W) or water-in-oil nanometric emulsions. Moreover, they can also be classified according to their droplet size, being divided into microemulsions (10–100 nm) or nanoemulsions (20–200 nm) (although the accepted size range can differ between authors). Aside from the possibility of having simple and fast preparation methods, nanometric emulsions also have all of the aforementioned biopharmaceutical advantages of nanosystems, and their lipophilic nature makes them ideal for solubilizing lipophilic drugs. They are also biocompatible, and the right formulas can be quite stable. Additionally, the surfactants and cosolvents that are part of these formulations composition have the ability to enhance drug permeation across biological barriers [11,15,16] (summary of these characteristics in Figure 2).

This review was projected with the purpose of collecting and analyzing detailed relevant information regarding nano- and micro-emulsions development for the delivery of antipsychotic drugs. This comprehensive information includes composition, droplet size, polydispersity index (PDI), zeta potential, viscosity, osmolarity/osmolality, pH, in vitro drug release, ex vivo drug permeation, in vivo pharmacokinetics, in vivo pharmacodynamics and/or safety studies, depending on the available original data. The final goal was to address what has been performed so far in this specific scientific pharmaceutical and medical field, while attempting to conclude on which nanometric emulsion composition and/or incorporated drug(s) could be most adequate for the therapeutic aim.

## 3. Nanometric Emulsions Containing Antipsychotic Drugs

Nanometric emulsions have been developed for the encapsulation of several different antipsychotic drugs, for parenteral, oral or intranasal administration routes. A detailed analysis of these topics is presented in the following Section 3.1, Section 3.2 and Section 3.3. A summary of the most relevant formulation parameters is depicted in Table 1.

### 3.1. First-Generation Antipsychotics

First-generation (or typical) antipsychotics are effective in attenuating schizophrenia’s positive symptoms, mainly associated with acute episodes, and preventing psychotic relapse [4].

Chlorpromazine was the first antipsychotic drug to ever be approved for clinical application, with its use spreading worldwide between 1952 and 1955 [38]. It is mostly a dopamine and serotonin receptor antagonist, but it also has affinity for other receptors, which leads to several severe systemic side effects, including agitation, convulsions, difficulty breathing and swallowing, extreme sleepiness, fever, irregular heart rate, and low blood pressure [17,39]. Its lipophilic nature leads to it evidencing both poor aqueous solubility (predictably 0.00417 mg/mL) and poor permeability, which in turn results in variable and low oral absorption. Moreover, chlorpromazine holds a high protein-binding and extensive first-pass metabolism. These characteristics are responsible of a low oral bioavailability [17,39]. To tackle these issues, Baloch et al. formulated chlorpromazine into a self-nanoemulsifying drug delivery system (SNEDDS). SNEDDS are mixtures of oils, surfactants and cosurfactants or cosolvents that spontaneously form a nanoemulsion when in contact with (diluted in) the gastrointestinal tract’s fluids [40]. All 3 formulas comprised Tween^®^ 85 (polysorbate 85-polyoxyethylene 20 sorbitan trioleate) as the hydrophilic surfactant and ethanol as cosolvent, and 2 of the formulas also had glycerol. The selected oil was different among the developed formulations, being either Triacetin (short-chain triglyceride), Captex^®^ 355 (medium-chain triglyceride, glyceryl tricaprylate/tricaprate) or olive oil + linseed oil (long-chain triglycerides, 1:2 *w*/*w*). Detailed quantities of each used ingredient are shown in Table 2. Drug strength was kept at 2% (*w*/*w*) for all formulations, which is 4796 times higher than chlorpromazine’s predicted aqueous solubility. SNEDDS’ droplet characteristics (after dilution) were 159–186 nm for droplet size, 0.27–0.33 for PDI, and −14.1 to −21.4 mV for zeta potential, with a pH between 7 and 7.5. Moreover, all SNEDDS were stable under accelerated conditions or storage for 3 months. Ex vivo permeation studies (rat intestine) showed that all SNEDDS had increased drug permeation when compared to a drug suspension, which was justified by the tight junctions’ opening effect of triglycerides that facilitates drug transport across the intestinal mucosa. Moreover the long-chain triglyceride formula showed the best performance, which suggests that the presence of oleic acid had a permeation enhancing effect. The in vivo pharmacokinetic studies (with rats; 2 mg drug/kg body weight) showed that the oral administration of all SNEDDS led to an at least 2-fold higher systemic bioavailability than the free drug suspension. The presence of a longer chain triglyceride seemed to improve the formulation’s performance once more, being responsible for the highest plasma area under the “drug concentration vs. time” curve (AUC) and maximum drug concentration (C_max_). Nevertheless, there was no intravenous control group, which would have provided important data regarding the drug’s systemic bioavailability. Moreover, the drug was never quantified in the animals’ brain, nor was a pharmacodynamic study performed, and hence the potential of the developed formulations to allow the drug to reach the brain and/or have a therapeutic effect was left undetermined.

Haloperidol is a strong dopamine D2 receptor antagonist that was approved for clinical use in the 1960s [18,41,42]. It is currently available in oral and injectable drug preparations, but it has limited uptake across the blood brain-barrier, and a vast distribution to non-targeted sites, which leads to quite severe side effects, such as movement disorders (dystonia, tardive dyskinesia, akathisia and drug-induced Parkinsonism), sedation, weight gain, prolactin changes and cardiac events. Moreover, haloperidol undergoes high liver metabolization, resulting in low oral bioavailability, and low aqueous solubility (predictably 0.00446 mg/mL) [18,42]. In order to overcome such limitations, El-Setouhy et al. developed O/W nanoemulsions for intranasal delivery, containing Capmul^®^ MCM (medium chain mono- and di-glycerides), Tween^®^ 80 (polysorbate 80-polyoxyethylene 20 sorbitan monooleate), Span 20 (sorbitan monolaurate) and Transcutol^®^ P (diethylene glycol monoethyl ether), prepared by spontaneous emulsification. Additionally, the mucoadhesive nanoemulsion also contained Pemulen™ TR-2 (acrylates/C10–30 alkyl acrylate crosspolymer) (specific quantity in Table 2). Obtained drug strength was 8.5 mg/mL, which is more than 1900 times higher than haloperidol’s aqueous solubility. For the selected non-mucoadhesive formula, droplet size was 209.5 nm, but PDI was 0.402, which is a reasonably high value, hence reflecting the non-homogeneous nature of the formulation, while zeta potential was found to be −23.85 mV. This same formula was stable after a 12-month storage period (both under refrigeration and at room temperature). However, the mucoadhesive nanoemulsion was not characterized, which raises the question of whether it was stable, homogeneous, and even if it was in fact a nanoemulsion. The in vivo pharmacokinetic study (mice; 2 mg/kg), which was only performed for the mucoadhesive nanoemulsion, (which was not the one that was previously characterized), showed that its intranasal administration led to a more effective and faster brain drug delivery (higher brain C_max_, with similar AUC, and lower “time to reach maximum drug concentration” (T_max_)), suggesting the superiority of this route of administration for this drug delivery purpose. Additionally, intranasal delivery led to brain/blood ratios that were higher than 1 at all time-points (which did not happen with the intravenous group), suggesting higher efficacy and safety (lower desirable systemic distribution). The fact that the drug was located within lipophilic nanodroplets might have facilitated the direct nasal transmucosal transport to the brain, in which the presence of the mucoadhesive Pemulen™ TR2 helped overcome nasal mucociliary clearance. Nevertheless, a mucoadhesive nanoemulsion is hardly an ideal intravenous control, especially if it is too viscous (an issue that was not addressed in this study), and the intravenous administration of a drug solution would have been a more adequate control, also providing a comparison with a different and simpler formulation. Additionally, the administration of the same formulation through two different routes did not allow us to understand whether the formulation itself was more effective or safer than others (drug solutions or suspensions), but only that its intranasal administration was more effective in making the drug reach the brain than the intravenous. Still, the in vivo pharmacodynamic study (locomotor activity) further confirmed the effectiveness of the developed intranasal mucoadhesive nanoemulsion, which was very effective in causing motor suppression. Safety evaluation (rabbits; 0.5 mg/kg) suggested that the developed formulation was also safe, with the animals’ nasal mucosa showing no histopathological alterations after chronic administration.

### 3.2. Second-Generation Antipsychotics

First-generation antipsychotics induce a variety of adverse events, including some severe neurological side effects, in both acute and long-term exposure. Moreover, these drugs are almost ineffective in the treatment of negative and cognitive symptoms. This led to a search for new drugs with higher efficacy and/or safety. Therefore, the second-generation, or “atypical”, antipsychotics were developed, being a series of molecules devoid of neurological side effects, with a generally improved safety profile, and with overall superior efficacy and (some) effectiveness against negative and/or cognitive symptoms [3,4].

Risperidone is one of the oldest second-generation antipsychotic drugs, having been approved for the treatment of schizophrenia in 1993, and acting as an inhibitor of dopaminergic D2 and serotonergic 5-HT2A receptors [19,43,44]. Nevertheless, its oral administration still leads to numerous side effects, such as dystonia, tremor, sedation, dizziness, and several gastrointestinal and upper respiratory tract symptoms, also displaying a slow onset of therapeutic action, which is especially relevant to counteract acute psychosis or emergency situations [20,21,22,44]. Moreover, risperidone has poor water solubility (predicted to be 0.171 mg/mL), and substantial first-pass metabolism and P-glycoprotein efflux [19,20,43]. To tackle these issues, and specifically for emergency situations and when medicine swallowing is not an option for administration, Dordevic et al. [19,20] developed O/W nanoemulsions for parenteral delivery. The oil mixture was made of medium chain triglycerides and soybean oil (Lipoid Purified Soybean Oil 700), and the formulation also contained soybean lecithin (Lipoid S 75), benzyl alcohol, butylhydroxytoluene, glycerol, sodium oleate and water (pH 9). Additionally, the formulations also comprised a hydrophilic surfactant, that was either Tween^®^ 80 (polysorbate 80-polyoxyethylene 20 sorbitan monooleate), Kolliphor^®^ P 188 (poloxamer 188) or Kolliphor^®^ HS 15 (macrogol 15 hydroxystearate) (detailed quantities in Table 3). The nanoemulsions were prepared by high pressure homogenization, and drug strength was set at 1 mg/g, which is almost 6 times higher than risperidone’s aqueous solubility. Measured droplet size was 160 nm, PDI between 0.10–0.13, zeta potential –50 mV, pH between 8.2–8.4, and viscosity between 6–11 cP (being considered sufficiently low for parenteral administration purposes), for all formulations. All preparations were stable after a 4-month storage period. The in vivo pharmacokinetic evaluation (rats; 1 mg/kg) indicated that the Tween^®^ 80-based nanoemulsion showed the best brain targeting profile, since it led to the highest brain C_max_ and AUC values when compared to the other nanoemulsions and a drug solution. Since all nanoemulsions had similar characterization parameters (droplet size, PDI, zeta potential, viscosity) and composition aside from the hydrophilic surfactant type, Tween^®^ 80 was indicated as the most suitable hydrophilic emulsifier for brain targeting purposes (between the ones that were studied), since it originated the highest brain drug levels. Therefore, in vivo pharmacodynamics (rats, locomotor activity assessment) was carried out using this formulation, which indeed had a more sustained antipsychotic effect, with less sedation (side-effect), when compared to a drug solution, further confirming its potential for risperidone brain delivery.

Kumar et al. [21,22] also decided on preparing O/W risperidone nanoemulsions, but in this case for intranasal administration. Formulations were prepared by spontaneous emulsification, with their composition including Capmul^®^ MCM (medium chain mono- and diglycerides), Tween^®^ 80 (polysorbate 80-polyoxyethylene 20 sorbitan monooleate), Transcutol^®^ P (diethylene glycol monoethyl ether), propylene glycol and water (specific quantities in Table 3). The mucoadhesive nanoemulsion also had chitosan. Drug strength was 3.5 mg/mL, which is more than 20 times higher than risperidone’s water solubility. Formulation characterization parameters were 14–18 nm for droplet size, 0.12–0.17 for PDI, −9 to −12 mV for zeta potential, 225–250 cP for viscosity, and 4.5–5.3 for pH. Osmolarity was approximately 270 mOsmol/L, which is within the established limits for intranasal preparations. Both formulations were stable after a 3-month storage and under accelerated conditions. In ex vivo drug permeation studies (sheep nasal mucosa), a risperidone solution (made of ethanol, propylene glycol and water) had the highest permeation coefficient, probably due to low viscosity and having cosolvents in its composition, followed by the mucoadhesive nanoemulsion, which performed better than the non-mucoadhesive nanoemulsion, probably due to the permeation enhancing properties of chitosan. In vivo pharmacokinetic studies (rats; 0.09 mg/kg) showed that the intranasal mucoadhesive nanoemulsion had the best brain drug targeting, since it had the highest brain C_max_ and AUC values, and also the highest brain/blood ratios at all time points, when compared to the other intranasal and intravenous formulations. Hence, chitosan not only had a role in enhancing drug permeation, but also in decreasing mucociliary clearance, with a clear consequence on brain bioavailability. To further confirm the potential of this formulation, in vivo pharmacodynamics (mice; 0.325 mg/kg; locomotor activity) indicated that both intranasal nanoemulsions performed better than the intravenous group, and between them the mucoadhesive nanoemulsion was superior, demonstrating higher brain drug uptake. Moreover, the formulation’s components did no damage to the nasal mucosa, as indicated by the nasal ciliotoxicity studies (sheep nasal mucosa).

Olanzapine is an atypical antipsychotic drug which was approved for clinical use by the American Food and Drug Administration (FDA) in 1996, acting as an antagonist of dopamine (mainly D2) and serotonin (mainly 5-HT2) receptors [23,45]. Its oral administration in tablet form leads to an extensive first-pass metabolism, with a slow onset of action [23,24]. Moreover, both oral and intramuscular marketed forms have been linked to adverse events such as somnolence, mydriasis, blurred vision and respiratory depression [24,45]. Since olanzapine is also a low water solubility drug (predicted to be 0.0942 mg/mL) [45], and in order to tackle some of its other issues, Kumar et al. [23] decided to formulate it into O/W nanoemulsions, for intranasal administration, containing Capmul^®^ MCM (medium chain mono- and diglycerides), Tween^®^ 80 (polysorbate 80-polyoxyethylene 20 sorbitan monooleate), ethanol, polyethylene glycol 400 and distilled water (detailed quantities in Table 4). Additionally, the mucoadhesive nanoemulsion also had chitosan. Formulations were prepared by spontaneous emulsification, and drug strength was kept at 8.5 mg/mL, more than 90 times higher than olanzapine’s water solubility. Droplet size varied between 20–24 nm, PDI between 0.25–0.30, zeta potential between −4 and −11 mV, viscosity between 0.10–0.13 cP, and pH between 4.5–5.7. The results of the in vivo pharmacokinetic study (rats; 0.225 mg/kg) showed that all intranasally administered formulations led to a lower brain T_max_ when compared to the intravenous control (non-mucoadhesive nanoemulsion), which suggested preferential nose-to-brain transport. Moreover, both intranasal nanoemulsions led to significantly higher brain drug levels and brain/blood ratios at all time points, when compared to the intravenous group and the intranasal solution (made of ethanol and propylene glycol). Furthermore, the mucoadhesive nanoemulsion led to higher brain C_max_ and AUC than the non-mucoadhesive nanoemulsion, which in turn had a better performance than the intranasal solution. This indicated that both the nanoemulsion’s general composition, specifically the excipients with permeation enhancing capability, and the mucoadhesive agent led to an increase in brain drug uptake. Nevertheless, as before, the intravenous control should have been a drug solution (for example, such as the one that was administered intranasally), since the pharmacokinetic profile of a mucoadhesive nanoemulsion given intravenously could be quite altered. The in vivo pharmacodynamic assessment (locomotor activity in mice, 0.325 mg/kg; and paw test in rats, 0.225 mg/kg) further confirmed the potential of the developed formulations, with the intranasal route showing superiority when compared to intravenous administration, and with the mucoadhesive nanoemulsion also having a better performance within the intranasally administered formulations.

Patel et al. [24] also chose to develop olanzapine O/W nanometric emulsions for intranasal delivery, containing oleic acid, Labrasol^®^ (polyethylene glycol-8 caprylic/capric glycerides), Kolliphor^®^ RH40 (polyoxyl 40 hydrogenated castor oil), Transcutol^®^ P (diethylene glycol monoethyl ether) and water (specific quantities in Table 4). The mucoadhesive microemulsion also had polycarbophil AA-1 in its composition. The formulations were prepared by spontaneous emulsification, and drug strength was 8 mg/mL, which is 85 times higher than olanzapine’s water solubility. Measured droplet sizes were 23.87 and 31.66 nm, PDI 0.121 and 0.251, zeta potential −35.14 and −42.15 mV, viscosity 75 and 93 cP, and pH 5.87 and 5.95, for the non-mucoadhesive and mucoadhesive microemulsions, respectively. The developed preparations were deemed stable after storage for 6 months and under accelerated conditions. The in vivo pharmacokinetic evaluation (rats; 0.16–0.21 mg/kg) showed that all intranasally administered formulations (microemulsions and propylene glycol drug solution) had higher brain/blood ratios when compared to the intravenous group (non-mucoadhesive microemulsion), which suggests a more favorable efficacy vs. safety profile for this administration route. Moreover, brain drug concentrations obtained with the intranasal microemulsions were significantly higher at all time points when compared with the intravenous group, with the intranasal microemulsions also having significantly higher brain C_max_ and AUC values, and lower T_max_, which indicates a faster and more effective brain drug delivery. Furthermore, the addition of the mucoadhesive polymer to the microemulsion led to higher brain drug uptake. Yet again, the intravenously administered formulation should have been a drug solution, and not a microemulsion, due to potentially altered pharmacokinetic profile and, in this specific case, a viscosity that is probably too high to be safely administered intravenously (without the risk of causing vascular adverse events). Nonetheless, the in vivo pharmacodynamics (mice, 3.20 mg/kg, apomorphine-induced compulsive behavior and spontaneous motor activity) confirmed an overall superiority of the intranasal route, and especially of the mucoadhesive microemulsion, in reversing induced schizophrenia-like symptoms, with the nanometric size, the presence of drug permeation enhancing excipients, and nasociliary clearance prevention by the mucoadhesive polymer being clearly important.

Quetiapine is another second-generation antipsychotic drug and was approved by the FDA in 1997 [46]. Its action is thought to be mainly mediated by dopamine D2 and serotonin 5-HT2 receptors antagonism [25,46]. Despite being considered to have an acceptable safety profile, it has been reported to cause somnolence, orthostatic hypotension and anticholinergic effects, suicidal thinking or behavior in children under 10 years of age, and an increased death rate in elderly patients with dementia [46]. Aside from side effects, this drug also has other issues, such as a short plasma half-life (hence requiring frequent administration), and low absorption and high metabolism after oral administration (leading to poor bioavailability) [25,26]. Moreover, it is a P-glycoprotein substrate, and is highly lipophilic (predicted water solubility 0.0403 mg/mL), also having a pH dependent solubility (higher solubility at lower pH) [25,26,46]. In order to solve some of these issues, Boche et al. [25] decided to develop an intranasal O/W nanoemulsion containing Capmul^®^ MCM (medium chain mono- and diglycerides), Tween^®^ 80 (polysorbate 80-polyoxyethylene 20 sorbitan monooleate), Transcutol^®^ P (diethylene glycol monoethyl ether), propylene glycol and water, with quetiapine in fumarate salt form (higher water solubility) (specific quantities in Table 5). Formulation preparation included a high sheer method (sonication), and measured droplet characteristics were 144 nm for droplet size, 0.193 for PDI, and −8.131 mV for zeta potential. The developed nanoemulsion was stable after a 6-month storage. The in vitro drug release assay (dialysis bag method) showed that the developed formulation had a more than 2-fold drug release increase when compared to a drug solution (water-ethanol). Moreover, the in vivo pharmacokinetic studies (rats; 1 mg/kg) indicated that the intranasal nanoemulsion had a better brain targeting, with higher brain C_max_ and AUC values, than the intranasal solution and both intravenous groups (solution and nanoemulsion). Moreover, brain T_max_ was lower for the intranasal groups, which means that intranasal delivery was faster in making the drug reach the brain. Additionally, brain/blood ratios were always equal to or higher than 1 for the intranasal nanoemulsion, which did not happen for any of the other groups, suggesting a higher efficacy vs. safety profile.

**Figure 3:**
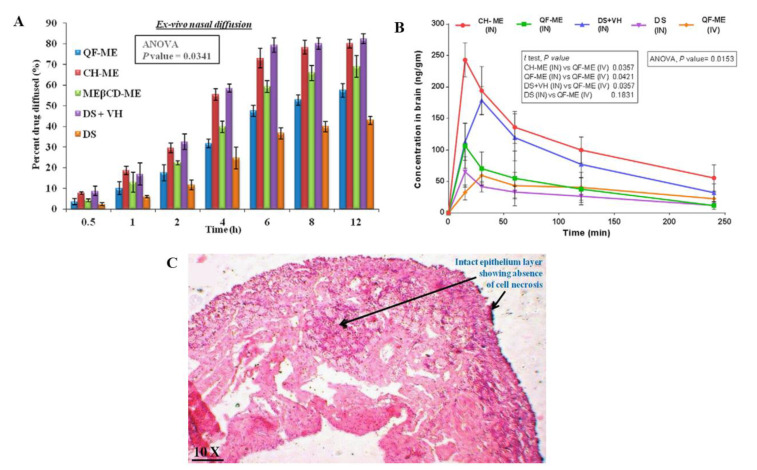
(**A**) Ex-vivo permeation results of quetiapine non-mucoadhesive microemulsion (QF-ME), mucoadhesive microemulsion (with chitosan, CH-ME), cyclodextrin microemulsion (with methyl-β-cyclodextrin, MeβCD-ME), and drug solution (DS); (**B**) brain drug concentration vs. time profiles after intranasal administration of quetiapine non-mucoadhesive microemulsion (QF-ME (IN)), mucoadhesive microemulsion (with chitosan, CH-ME (IN)) and drug solution (DS (IN)), and intravenous administration of non-mucoadhesive microemulsion (QF-ME (IV)) to rats; (**C**) histopathological examination of a goat nasal mucosa section after exposure to the quetiapine mucoadhesive microemulsion; adapted from Shah et al. [26], reproduced with permission from Elsevier [License Number 5393031395763].

Shah et al. [26] also developed quetiapine O/W nanometric emulsions, for intranasal administration, using Capmul^®^ MCM (medium chain mono- and diglycerides), Labrasol^®^ (polyethylene glycol-8 caprylic/capric glycerides), Tween^®^ 80 (polysorbate 80-polyoxyethylene 20 sorbitan monooleate), Transcutol^®^ P (diethylene glycol monoethyl ether) and water (pH 5) (detailed amounts in Table 5), but this time the preparation method was spontaneous emulsification. Drug strength was kept at 6 mg/mL, which is 149 times higher than quetiapine’s water solubility. Three formulas were developed, one being a non-mucoadhesive microemulsion, another being a mucoadhesive microemulsion (additionally having chitosan), and a third one having cyclodextrins (trimethyl-beta-cyclodextrins). Measured droplet sizes were between 29.75–46.55 nm, PDI between 0.221–0.249, zeta potential between 2.77–20.29 mV, viscosity between 17.5–38.5 cP, and pH between 5.5–6.5 (safe for the human nasal mucosa). All formulations were stable under accelerated conditions. The ex vivo mucoadhesive strength test (goat nasal mucosa) showed that both the mucoadhesive microemulsion and the microemulsion with cyclodextrins had an improved adhesion to the mucosal surface (although the mucoadhesive formulation had a more substantial improvement). This could be attributed to chitosan having an ionizable positively charged group, capable of interacting with the negatively charged groups of sialic acid on the mucosal surface, and cyclodextrins also interacting with the same residues, but by hydrogen bond formation. Ex vivo drug permeation (goat nasal mucosa, Figure 3A) was highest for the mucoadhesive microemulsion, when compared to the other microemulsions, and all microemulsions had an increased drug permeation when compared to a drug solution. The superiority of mucoadhesive microemulsion performance could be attributed to the presence of chitosan and its reported ability to enhance paracellular transport by disruption of tight junctions. In vivo pharmacokinetics (rats, 2.3 mg/kg, Figure 3B) further confirmed the superiority of the mucoadhesive microemulsion, since the intranasal administration of this formulation led to quetiapine brain concentrations that were significantly higher at all the time points when compared to all other groups (intranasal and intravenous), also having higher brain C_max_ and AUC values, and brain/blood ratios. Moreover, intranasal administration led to overall lower plasma drug levels when compared to the intravenous group which could indicate a higher safety profile (although, again the intravenously administered formulation should have been a drug solution instead of the non-mucoadhesive microemulsion). As for formulation safety (nasal ciliotoxicity in goat nasal mucosa, Figure 3C), the mucoadhesive microemulsion showed no signs of cell necrosis or structural damage, indicating it to be apparently safe for intranasal administration.

Ziprasidone is a dopamine D2 and serotonin 5-HT2A receptor antagonist and was approved by the FDA for the treatment of schizophrenia in 2001 [27,47,48]. It is commercialized as an oral capsule but has low bioavailability due extensive first-pass metabolism, and also leads to several systemic side effects (such as somnolence, respiratory tract infections, dizziness, akathisia, abnormal vision and asthenia). Moreover, its water solubility is quite low (predicted to be 0.00718 mg/mL), which poses a problem for the formulation process [27,48]. In order to solve some of these issues, Bahadur et al. [27] decided to develop O/W nanoemulsions for intranasal delivery. The formulations contained Capmul^®^ MCM (medium chain mono- and diglycerides), Labrasol^®^ (polyethylene glycol-8 caprylic/capric glycerides), Transcutol^®^ (diethylene glycol monoethyl ether) and phosphate buffer (pH 8.0) (specific quantities in Table 5). Additionally, the mucoadhesive nanoemulsions also had either chitosan or Carbopol^®^ 934 (carbomer 934) in their composition. Drug strength was set at 20 mg/mL, which is 2786 times higher than ziprasidone’s water solubility, and formulations were prepared by spontaneous emulsification. Mean droplet size, PDI, zeta potential, pH and viscosity of the non-mucoadhesive nanoemulsion were 145.24 nm, 0.186, −30.2 mV, 6.5 and 183 cP, respectively. This same formulation was considered to be stable under accelerated conditions. Nevertheless, these characterization and stability parameters were not measured for the mucoadhesive nanoemulsions, which leaves the question of whether these formulations were actually nanoemulsions, since the addition of a mucoadhesive polymer to a nanoemulsion can alter droplet size and PDI greatly, as well as the formulation’s viscosity (which the authors refer is an important parameter but do not measure for the mucoadhesive formulations). All formulations had an osmolarity of around 310 mOsmol/L, which is considered safe for the nasal mucosa. The ex vivo permeation assay (sheep nasal mucosa) indicated that the mucoadhesive nanoemulsions had higher diffusion coefficients (when compared to the non-mucoadhesive nanoemulsion), with the one containing chitosan having a better performance than the one containing Carbopol, which might be explained by chitosan’s capacity for permeation enhancing due to reversible opening of tight junctions (as previously mentioned) or modifying phospholipids bilayers. In vivo pharmacodynamic results (mice, locomotor activity) indicated that, in general, the intranasal administration of the developed nanoemulsions led to a more significant therapeutic effect than oral or intraperitoneal administrations, suggesting the superiority of intranasal delivery. Moreover, the mucoadhesive chitosan nanoemulsion had the best performance, which again could be due to chitosan not only increasing the residence time of the formulation in the nasal mucosa but also enhancing drug permeation. Nevertheless, again the intraperitoneally administered formulation should have been a drug solution, and it was the non-mucoadhesive nanoemulsion, which could have led to altered pharmacokinetics, also being a highly viscous preparation (which should not be given through this route). Yet, nasal ciliotoxicity studies (sheep nasal mucosa) showed that the developed nanoemulsions could be regarded as safe, since they did not cause any sort of acute damage to the nasal mucosa.

Paliperidone is another second-generation atypical antipsychotic and was approved by the FDA for the treatment of schizophrenia in 2006 [49]. It is the primary active metabolite of risperidone (9-hydroxyrisperidone), and acts by blocking dopamine D2 and serotonin 5-HT2A receptors. Nevertheless, its oral bioavailability (extended-release tablet) is only 28%, and has low water solubility (predicted to be 0.297 mg/mL), making it hard to formulate at high strength [28,49]. Hence, Patel et al. [28] decided to incorporate paliperidone into O/W microemulsions for intranasal administration. Both microemulsions had oleic acid, Labrasol^®^ (polyethylene glycol-8 caprylic/capric glycerides), Kolliphor^®^ RH40 (polyoxyl 40 hydrogenated castor oil), Transcutol^®^ P (diethylene glycol monoethyl ether) and water, and the mucoadhesive microemulsion additionally had polycarbophil AA-1 (detailed quantities in Table 6). The chosen preparation method was spontaneous emulsification, and drug strength was 5 mg/mL, which is almost 17 times higher than paliperidone’s water solubility. Formulation characterization parameters were between 20.01–27.31 nm for droplet size, 0.049–0.117 for PDI, −36.59 and −38.65 mV for zeta potential, 77–96 cP for viscosity, and 5.85–5.98 for pH. The developed formulations were stable up to 6 months and under accelerated conditions. The in vivo pharmacokinetic study (rats; 0.18–0.20 mg/kg) indicated that, overall, the intranasal route was faster than the intravenous route at making the drug reach the brain (lower T_max_ for the non-mucoadhesive microemulsion). Moreover, both microemulsions were more effective in delivering the drug to the brain, having higher brain C_max_, brain AUC and brain/blood ratio values than the intravenous group, with the mucoadhesive microemulsion having a better performance. Yet again, the intravenous control should not have been a microemulsion, especially since it is reasonably viscous. In vivo pharmacodynamics (mice; 0.20 mg/kg; apomorphine-induced compulsive behavior and spontaneous motor activity) confirmed the overall potential of the intranasal route, and especially of the developed microemulsions, with the mucoadhesive polymer clearly having an important role and resulting in higher therapeutic efficacy.

Pidaparthi et al. [29] also formulated paliperidone into an O/W nanometric emulsion for intranasal delivery. The nanoemulsion’s composition included Labrafil^®^ M 1944 CS (oleoyl polyoxyl-6 glyceride), Tween^®^ 80 (polysorbate 80-polyoxyethylene 20 sorbitan monooleate), Transcutol^®^ HP (diethylene glycol monoethyl ether), benzalkonium chloride and phosphate buffer (pH 6.4) (specific amounts in Table 6). The preparation method was water titration followed by high-pressure homogenization, and measured characterization parameters were −0.009 mV for zeta potential, 6.5 for pH, 94.5 cP for viscosity and 38.25 nm for droplet size, with no PDI reported, which leaves a question about the formulation’s homogeneity. Drug strength was kept at 2.5 mg/mL, which is more than 8 times higher than paliperidone’s water solubility, and the developed nanoemulsion was stable under accelerated conditions. In vitro drug release (Franz diffusion apparatus) and ex vivo drug permeation (goat nasal mucosal) studies showed that the paliperidone nanoemulsion had a more than 4 times higher drug release and a more than twice higher drug permeation than a drug suspension. As for in vivo evaluation, pharmacodynamic studies (mice; 2 mg/kg; spontaneous motor activity) showed that the intranasal administration of the paliperidone nanoemulsion led to a more effective therapeutic action than the intranasal drug suspension. No other controls were carried out, but it would have been interesting to have an intravenous control group (drug solution), where the drug would have been readily systemically available. The developed formulation was also safe, given that in continuous intranasal administration to mice (every 3 h for 12 h), their nasal mucosa showed no signs of inflammation, hemorrhage, erosion or nasociliary damage.

Iloperidone is another atypical antipsychotic drug that acts as an antagonist for both dopamine D2 and serotonin 5-HT2 receptors, having been approved for schizophrenia treatment by the FDA in 2009. It is available as oral tablets, but extensive first-pass metabolism leads to low bioavailability, and it also has poor water solubility (predictably 0.0304 mg/mL) [30,50]. To help solve these issues, Narala et al. developed an O/W nanoemulsion, for oral administration, containing soybean oil (Lipoid), egg lecithin (egg phosphatidyl choline, EPC80), cholesterol, oleic acid, glycerol and water (detailed quantities in Table 6). The formulation was prepared by hot homogenization and ultrasonication, and drug strength was 0.4 mg/mL, the equivalent to 13 times iloperidone’s water solubility. The measured droplet size was 222.3 nm, PDI 0.200, and zeta potential −28.9 mV, and stability studies deemed the developed nanoemulsion as fairly stable after storage for 3 months and under accelerated conditions. In vitro drug release (open tube dialysis method) appeared to be sustained, being below 50% (cumulative release) after 12 h, and only being close to full release after 48 h (approximately 90% cumulative release). Oral administration to rats (2.5 mg/kg) led to a higher systemic bioavailability than an oral drug suspension (higher plasma C_max_ and AUC), and also to a higher suppression of hyperlocomotor activity (pharmacokinetic and pharmacodynamic studies, respectively). Nevertheless, the comparison to an intravenous control (especially if it was a drug solution) would have been useful.

**Figure 4:**
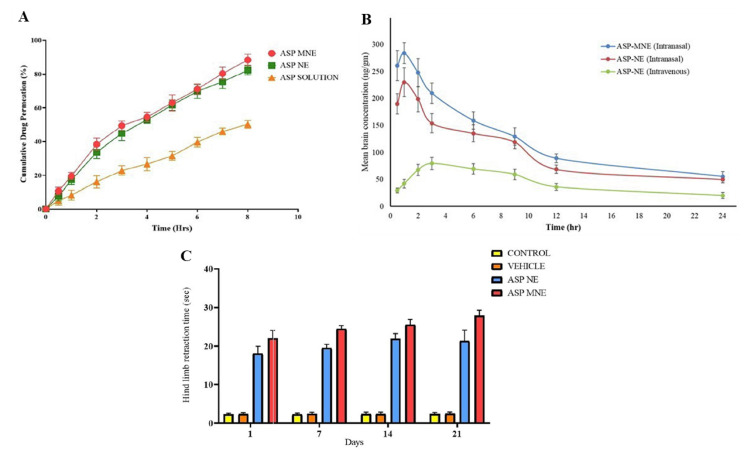
(**A**) Ex vivo drug permeation results of asenapine non-mucoadhesive nanoemulsion (ASP NE), mucoadhesive nanoemulsion (ASP MNE) and drug solution (ASP SOLUTION); (**B**) brain pharmacokinetic profile of asenapine non-mucoadhesive nanoemulsion (ASP-NE (Intranasal)) and mucoadhesive nanoemulsion (ASP MNE (Intranasal)) after intranasal administration, and non-mucoadhesive nanoemulsion (ASP-NE (Intravenous)) after intravenous administration; (**C**) results of the hind-limb retraction response (paw test) after chronic intranasal administration of asenapine non-mucoadhesive nanoemulsion (ASP NE) and mucoadhesive nanoemulsion (ASP MNE) (compared to control and vehicle); adapted from Kumbhar et al. [31], reproduced with permission from Elsevier [License Number 5393031097560].

Asenapine is a dopamine D2 and serotonin 5-HT2A receptors antagonist and was approved for the treatment of schizophrenia by the FDA in 2009. After oral administration it leads to a very low bioavailability, having extensive first-pass metabolism and low water solubility (predicted to be 0.0312 mg/mL) [31,51]. To address these issues, Kumbhar et al. [31] developed O/W nanoemulsions for intranasal administration, containing Capmul^®^ PG-8 (propylene glycol monocaprylate), Kolliphor^®^ RH40 (polyoxyl 40 hydrogenated castor oil) and Transcutol^®^ HP (diethylene glycol monoethyl ether). Moreover, the mucoadhesive microemulsion also had Carbopol^®^ 971 (carbomer 971) in its composition (specific quantities in Table 6). The non-mucoadhesive nanoemulsion had a droplet size of 21.2 nm, PDI of 0.355, zeta potential of −14.12 mV and viscosity of 91.67 cP. Aside from viscosity, the mucoadhesive microemulsion did not have these parameters determined, which again leaves the question of whether the formulation obtained after incorporation of the mucoadhesive polymer was still a microemulsion, and, if so, how homogeneous it was. The mucoadhesive microemulsion’s viscosity was 235.67 cP (higher, as expected from the addition of Carbopol). The formulations were prepared by aqueous titration and were stable under accelerated conditions. The in vitro drug release (Franz diffusion cells) from the developed nanoemulsions was higher than from an asenapine solution, and the mucoadhesive formulation, being more viscous, had a slightly lower cumulative drug release than the non-mucoadhesive nanoemulsion. Nevertheless, the ex vivo permeation assay (sheep nasal mucosa, Figure 4A) showed that the mucoadhesive nanoemulsion had a higher overall drug permeation, which might, again, be due to transient opening of the tight junctions of the nasal mucosa due to the presence of the mucoadhesive Carbopol. Furthermore, the nanoemulsion’s mucoadhesive strength (ex vivo assay in sheep nasal mucosa) increased more than 3-fold with the addition of the mucoadhesive polymer. In vivo pharmacokinetic results (rats, 1 mg/kg, Figure 4B) indicated the superiority of intranasal administration, since both intranasal nanoemulsions had significantly higher brain C_max_ and brain AUC and a lower brain T_max_ than the intravenous administration, suggesting that intranasal delivery was both faster and more effective in making the drug reach the brain. Moreover, the intranasal mucoadhesive nanoemulsion had a better performance than its non-mucoadhesive counterpart, probably due to enhanced viscosity and adhesion to the nasal mucosa, which resulted in prolonged retention time in the nasal cavity. Nevertheless, again, the intravenous control group should have probably been a drug solution, given the more predictable pharmacokinetics and lower viscosity. In vivo pharmacodynamics (rats; 1 mg/kg; catalepsy test, induced locomotor activity test and paw test–Figure 4C) indicated that the developed nanoemulsions had therapeutic-like efficacy, showing better results that the drug solution, and with the mucoadhesive formulation performing better. As for the formulations’ safety (nasal ciliotoxicity, sheep nasal mucosa), they did not seem to cause morphological changes or other signs of toxicity in the nasal mucosa and were thus considered safe.

Amisulpride is another atypical antipsychotic drug which is not only highly selective (antagonist of dopamine D2 and D3 receptors), which decreases the likelihood of extrapyramidal symptoms, but also has the rare ability of reasonably improving both positive and negative symptoms of schizophrenia. Nevertheless, it has low intestinal permeability, which leads to low oral bioavailability, and is a P-glycoprotein substrate. Moreover, it has low aqueous solubility (predicted to be 0.293 mg/mL), which is also pH dependent, resulting in high solubility in the stomach (low pH) but poor solubility in the intestinal medium (higher pH), which could cause drug precipitation when reaching the intestine after oral administration [32,52]. Moreover, albeit selective, there have been reports of cardiovascular (prolongation of QT interval, bradycardia, hypotension), neuropsychiatric (sedation, seizures) and endocrine (increased prolactin levels) adverse events, as well as hematological toxicity with prolonged therapy (agranulocytosis or leukopenia) [33,52]. To solve some of these problems, Gamal et al. [32] decided to develop an oral SNEDDS, made of Capryol™ 90 (propylene glycol monocaprylate (type II)), Cremophor^®^ RH40 (polyoxyl 40 hydrogenated castor oil) and Transcutol^®^ HP (diethylene glycol monoethyl ether), with varying proportions (detailed quantities in Table 7). All 3 selected formulas had a drug strength of 50 mg/g, which is 170 times higher than amisulpride’s predicted water solubility. Formulations’ measured droplet size was between 16–22 nm, PDI between 0.092–0.193, and all formulas were stable under accelerated conditions. In vitro drug release (dialysis bag method) in a low pH medium (1.2) showed no statistically significant differences between the developed formulations and an aqueous drug suspension, but at neutral pH (7.4) there was a significantly higher amisulpride release from the developed SNEDDS, which suggests that these formulations could be useful in preventing drug precipitation at intestinal conditions. Drug permeation (ex vivo, rat intestine) was also significantly higher with the developed SNEDDS (compared to the drug solution), which shows the permeation enhancing effects of these formulation’s excipients and its favorable properties after dilution (nanometric droplet size). However, no in vivo evaluation was carried out, and hence the true potential of the developed SNEDDS was left undetermined.

**Figure 5:**
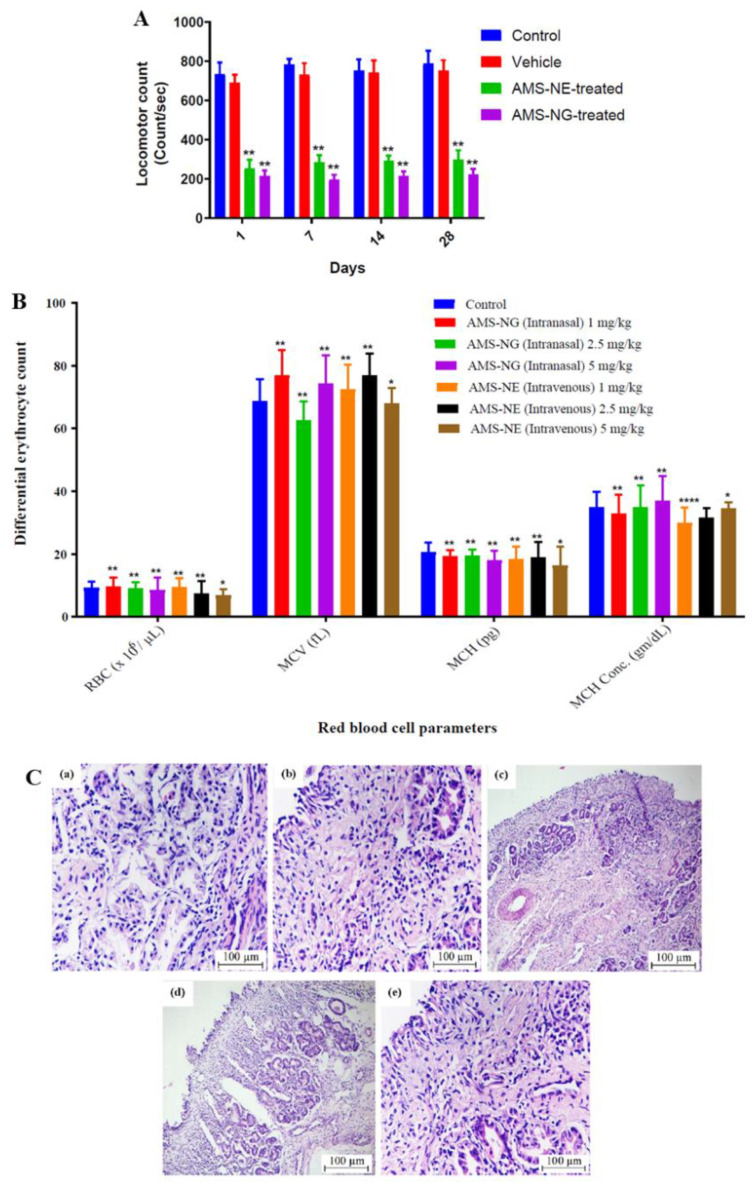
(**A**) locomotor activity after chronic intranasal administration of amisulpride nanoemulgel (AMS-NG-treated) or nanoemulsion (AMS-NE-treated) (compared to control and vehicle); (**B**) hematological toxicity (differential erythrocytes parameter counts) after intranasal administration of the amisulpride nanoemulgel (AMS-NG (Intranasal)) and intravenous administration of the nanoemulsion (AMS-NE (Intravenous)), at several doses; (**C**) histopathological examination of a rat nasal mucosal section after chronic exposure to several doses of the amisulpride nanoemulgel (**b**–**d**) (compared to control (**a**), and vehicle (**e**)); adapted from Gadhave et al. [33], reproduced with permission from Elsevier [License Number 5393040123016]. * *p* < 0.05, ** *p* < 0.01, **** *p* < 0.0001 vs. control group.

Gadhave et al. [33] also developed amisulpride formulations, but for intranasal delivery. The developed nanoemulsions had Maisine^®^ CC (glyceryl monolinoleate), Labrasol^®^ (polyethylene glycol-8 caprylic/capric glycerides), Transcutol^®^ HP (diethylene glycol monoethyl ether) and water in their composition (concentration of each component in Table 7). One of them also had poloxamer 407 and gellan gum (combination of thermosensitive and ion-sensitive polymers), making it a nanoemulgel. Nanoemulgels are nanoemulsions that have a gelling (and sometimes mucoadhesive) polymer in their composition, which gives them a higher viscosity that can increase their retention time in the nasal mucosa, consequently increasing the time available for drug absorption to occur. An aqueous-titration technique was the chosen preparation method, and drug strength was kept at 1 mg/mL, which is more than 3 times higher than amisulpride’s predicted water solubility. Measured formulation characteristics were 92.15 and 106.11 nm for droplet size, 0.46 and 0.51 for PDI, and 18.22 and –16.01 mV for zeta potential, for the non-mucoadhesive and mucoadhesive nanoemulsions, respectively. Although the other parameters could be considered to be within acceptable values, the PDI values were reasonably high, and hence the homogeneity of the developed nanometric emulsions could be questioned. Viscosity values were 8.69 cP (nanoemulsion) and 12.6 cP (nanoemulgel) at room temperature (25 °C), but at nasal temperature (34 °C) the viscosity of the nanoemulgel increased to 106.5 cP, due to having poloxamer 407 in its composition (thermosensitive polymer). The incorporation of the mucoadhesive polymer into formulation’s composition (gellan gum) also led to an increase in the mucoadhesive force (sheep nasal mucosa). Both formulas were stable under accelerated conditions. Compared to an amisulpride drug suspension (with 2% hydroxypropyl methylcellulose and 4% Tween^®^ 80), the developed formulations had a higher in vitro drug release (vertical Franz diffusion cell), and despite having a higher viscosity the nanoemulgel did not have a decrease in performance (when compared to the non-mucoadhesive nanoemulsion) but had a sustained-release effect (after 8 h). Additionally, ex vivo drug permeation (sheep nasal mucosa) led to a significantly higher cumulative permeation (also after 8 h) for the nanoemulgel, when compared to the nanoemulsion and the suspension, which indicates the advantage of not only having excipients with permeation enhancing capability (surfactants and cosolvents) and an increased surface area (nanodroplets), but also of the presence of the chosen polymers. In vivo pharmacokinetic studies (rats; 1 mg/kg) showed that the intranasal delivery of the developed formulations was better at making the drug reach the brain than the intravenous route, with the nanoemulgel having the highest brain drug delivery and least systemic drug distribution (leading to the most favorable brain/blood ratios and, consequently, a potentially higher efficacy and safety). In vivo pharmacodynamics also proved that the developed formulations were effective in reducing schizophrenia symptoms (catalepsy test, induced locomotor activity—Figure 5A, paw test), with the nanoemulgel having a better performance. This could have been due to the mucoadhesive properties and in situ gelling of the nanoemulgel, which made it possible for the formulation to have a longer residence time in the nasal cavity, leading to a longer time for drug absorption to occur. Nevertheless, again the intravenous control should have been a drug solution, and not the developed nanoemulsion. As for safety evaluation (rats, chronic administration for 28 days), the intravenous administration led to nasal or respiratory toxicity, and behavioral changes, while the intranasal administration did not (Figure 5C). Moreover, and the intranasal nanoemulgel led to a much lower hematological toxicity (Figure 5B) than the nanoemulsion, which further emphasizes the potential of the intranasal delivery of the developed nanoemulgel.

Lurasidone is another second-generation antipsychotic, having been approved by the FDA for the treatment of schizophrenia in 2010 [53]. It acts as an antagonist of dopamine D2 and serotonin 5-HT2A receptors, being a potent drug and having only a weak affinity towards other receptors, hence leading to less extrapyramidal side effects and a reasonably good tolerability profile. Yet, there have still been reports of systemic side effects (elevated levels of serum prolactin, orthostatic hypotension, muscle tremor), and due to having low water solubility (predictably 0.00789 mg/mL) and being highly metabolized, lurasidone also has low oral bioavailability (although improved when taken with food) [34,53]. To improve at least some of these features, Patel et al. [34] decided to formulate lurasidone into O/W nanoemulsions for intranasal administration. Their composition included Capmul^®^ MCM EP (medium chain mono- and diglycerides), Capmul^®^ MCM C8 EP (glyceryl monocaprylate), Cremophor^®^ EL (polyoxyl 35 castor oil) and water (detailed quantities in Table 7). The mucoadhesive nanoemulsion also had polycarbophil AA-1, and the chosen preparation method was high pressure homogenization. Drug strength was kept at 10 mg/mL, which is 1267 times higher than lurasidone’s predicted water solubility. The non-mucoadhesive nanoemulsion had a droplet size of 48.07 nm and PDI of 0.31, but these parameters were not mentioned for the mucoadhesive formulation, which leaves it unclear as to whether it was in fact nanometric or homogeneous. For both formulations, zeta potential varied between −0.19 and −0.41 mV and pH between 5–6. The incorporation of the mucoadhesive polymer into the nanoemulsion made the viscosity increase 8-fold (from 119.46 to 892.33 cP), and the retention time (mucoadhesion assay) 4-fold (5 min to 21 min). The formulations were stable under accelerated conditions and after storage for 6 months. Ex vivo permeation studies (sheep nasal mucosa) showed that the developed nanoemulsions led to a more effective and faster drug permeation than a drug solution (water:Transcutol^®^ P mixture 1:1 *v*/*v*), with the mucoadhesive nanoemulsion having a more sustained release due to being more viscous. Nevertheless, they did not perform better than a microemulsion (containing Capmul^®^ MCM EP, Labrasol^®^, Cremophor^®^ RH 40, Transcutol^®^ P and water), that was also evaluated for comparison purposes, probably due to this microemulsion having a higher amount of permeation enhancing excipients (surfactants and cosolvents), but which also make it potentially less safe. In vivo pharmacodynamic evaluation (mice; 4.06 mg/kg; apomorphine induced compulsive behavior and spontaneous locomotor activity) showed that intranasal administration was, overall, more therapeutically effective than the intravenous one (although a nanoemulsion was, again, the intravenous control, and not a drug solution), and that the intranasal nano and microemulsions performed better than the intranasal drug solution, despite the solution having a cosolvent in its composition. This could be due to the high surface area ratio that the nanometric emulsions provide, and also the presence of surfactants. Moreover, the mucoadhesive nanoemulsion performed better than the non-mucoadhesive nanoemulsion, which suggests that the mucoadhesive polymer did in fact increase the formulation’s retention time in the nasal mucosa, decreasing and/or slowing down the nasociliary clearance. Nevertheless, the microemulsion still had the highest therapeutic effect, again probably due to the higher amount of cosolvents, which enhanced drug absorption and, consequently, brain bioavailability. As for safety evaluation (nasal ciliotoxicity, sheep nasal mucosa), the developed formulations did not appear to damage the nasal mucosa, hence being potentially safe for intranasal administration.

### 3.3. Third-Generation Antipsychotics

Despite being generally safer than first-generation drugs, second-generation antipsychotics can still lead to a series of cardiovascular, cerebrovascular and metabolic adverse events, which might lead to treatment discontinuation [3]. Therefore, molecules with a higher selectivity for receptors that are linked to therapeutic activity should decrease the incidence of adverse events and, consequently, increase treatment adherence. Third-generation antipsychotics were designed to have these characteristics.

Aripiprazole was approved by the FDA in 2002, and is an atypical third-generation antipsychotic that, opposed to most other antipsychotic drugs, acts as a partial agonist for dopamine D2 receptors and agonist of serotonin 5-HT1A receptors, being reported to be effective against both positive and negative symptoms of schizophrenia [36,54]. Howver, although it has been considered to be comparatively safer than other similar therapeutics, there have been reports of serious systemic side effects (hypotension, hyperglycemia, neuroleptic malignant syndrome, QT interval prolongation), and it has a very low water solubility (predictably 0.00777 mg/mL), which makes it challenging to formulate at high strength [35,36,37,54]. In order to tackle these issues, Samiun et al. and Masoumi et al. [35,36] formulated aripiprazole into an O/W nanoemulsion, for intravenous administration. The nanoemulsion was prepared using high energy emulsification methods, and contained palm kernel oil esters, soybean lecithin (Lipoid S75), Tween^®^ 80 (polysorbate 80-polyoxyethylene 20 sorbitan monooleate), glycerol and water (specific quantities in Table 8). The drug was kept at 0.10 *w*/*w*%, which is 129 times higher than aripiprazole’s aqueous solubility. As for formulation characterization parameters, droplet size was around 60 nm, PDI 0.18, zeta potential −31.6 mV, viscosity 3.72 cP, osmolality 297 mOsm/kg and pH 7.4. Stability was assessed under accelerated conditions and after 3 to 9 months storage, and the results were favorable. Nevertheless, despite the successful development of this formulation, no further studies were conducted, and hence the true potential of this aripiprazole nanoemulsion (especially its efficacy in delivering the drug to the brain) was left undetermined.

**Figure 6:**
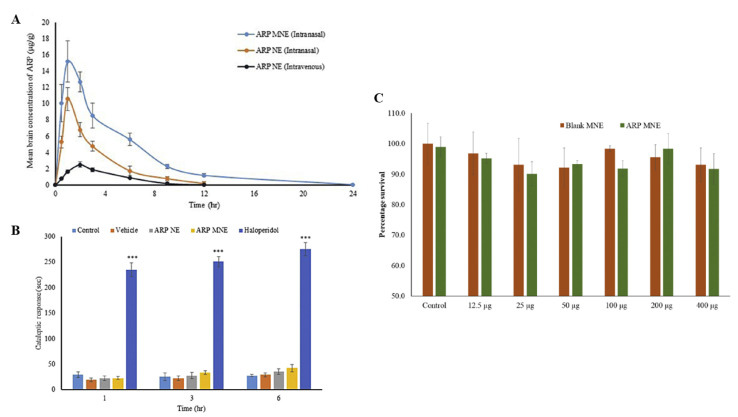
(**A**) Brain drug concentration vs. time profile after intranasal administration of aripiprazole non-mucoadhesive nanoemulsion (ARP NE (Intranasal)) and mucoadhesive nanoemulsion (ARP MNE (Intranasal)), and after intravenous administration of the non-mucoadhesive nanoemulsion (ARP NE (Intravenous)); (**B**) cataleptic response on the 21st day after intranasal administration of the aripiprazole non-mucoadhesive nanoemulsion (ARP NE) and mucoadhesive nanoemulsion (ARP MNE) (compared to vehicle and controls); (**C**) cytotoxicity results after vero cell exposure to the aripiprazole mucoadhesive nanoemulsion (ARP MNE) and blank vehicle (blank MNE); adapted from Kumbhar et al. [37], reproduced with permission from Elsevier [License Number 5393030780780]. *** *p* < 0.001 vs. control group.

Kumbhar et al. [37] also developed aripiprazole O/W nanoemulsions, but for intranasal administration, containing Capmul^®^ PG-8 (propylene glycol monocaprylate), D-α tocopheryl polyethylene glycol 1000 succinate (TPGS), Transcutol^®^ HP (diethylene glycol monoethyl ether) and water (Table 8). The mucoadhesive nanoemulsion also had Carbopol^®^ 971 (carbomer 971) in its composition. The chosen preparation method was spontaneous emulsification and the obtained droplet sizes were between 120–140 nm, PDI between 0.250–0.400 (this last value a little too high), and zeta potential between −16 and −19 mV (for both mucoadhesive and non-mucoadhesive nanoemulsions). The viscosity of the non-mucoadhesive nanoemulsion was also measured, being equal to 89.34 cP, (although the viscosity of the mucoadhesive formulation should also have been measured). The formulations were stable under accelerated conditions. In vitro drug release (Franz diffusion cells) showed that the cumulative percentage was approximately between 85 and 89% (8 h), with the mucoadhesive nanoemulsion being slightly slower, which might be explained by the existence of a polymer barrier (Carbopol) which hinders drug diffusion. Nevertheless, ex vivo drug permeation (sheep nasal mucosa) with the mucoadhesive nanoemulsion was higher than with the non-mucoadhesive nanoemulsion, and both formulations were better when compared to a drug solution, which not only shows the superiority of the nanoformulations, but also indicates that there is a positive influence of the presence of Carbopol, (again) probably by opening the tight junctions and enhancing paracelular drug transport. The mucoadhesive strength test (sheep nasal mucosa) also showed that there was an improved bioadhesion to the nasal mucosa with the incorporation of Carbopol into the formulation. Furthermore, the in vivo pharmacokinetic study (rats; 2 mg/kg; Figure 6A) not only confirmed the superiority of the intranasal route (lower brain T_max_ and higher C_max_ and AUC) when compared to intravenous administration (non-mucoadhesive nanoemulsion, again, not ideal), but also showed that the mucoadhesive nanoemulsion had the highest brain C_max_ and AUC of the two nanoemulsions, which shows that the mucoadhesive polymer increased the retention time of the formulation in the nasal cavity, thereby leading to an enhanced absorption. Moreover, the developed nanoemulsions were also effective in reversing schizophrenia-like symptoms in an animal model after chronic administration (rats; 1 mg/kg for 21 days; cataleptic test—Figure 6B, induced locomotor activity and paw test), with the mucoadhesive nanoemulsion being overall superior. These formulations also appeared to be safe, since results from in vitro (cytotoxicity, kidney cells, Figure 6C) and ex vivo (ciliotoxicity, sheep nasal mucosa) studies showed no significant changes when compared to the controls.

## 4. Authors’ Opinion

The majority of the studies included in this review were focused on the development of nanometric emulsions containing antipsychotics for intranasal administration. Intranasal administration has received increased attention over the years, especially for brain drug targeting [11], mostly due to its unique advantages when compared to other routes. Firstly, it allows for (at least part of) the drug to reach the brain directly by neuronal transport, which is ideal for affections with a brain etiology. This is usually associated with a reduction in systemic side effects (reduction in systemic distribution), increased therapeutic efficacy (higher brain bioavailability), and faster therapeutic effect (shorter onset of action). Additionally, it also allows (at least part of) the drug to avoid the blood-brain barrier and gastrointestinal and hepatic degradation, is non-invasive, and can be easily used in self-administration. All these particular features make this route potentially suitable for both chronic and emergency situations [7,11,55]. Another non-invasive and quite commonly used administration route is the oral route. Oral formulations are usually easy to self-administer, painless and cost-effective. Additionally, when administered through the oral route, drugs have access to a large surface area available for systemic absorption [56,57,58]. Within the studies included in this review, 14% of the developed formulations were designed for oral administration. The remaining 19% of articles developed formulations were aimed for parenteral administration. Albeit being an invasive route of administration (with all the well-known associated disadvantages), parenteral administration involves the direct injection of drugs into the bloodstream, which leads to the fastest systemic delivery with the highest bioavailability, since it allows avoiding all kinds of physical, chemical or biological barriers that could hinder drug absorption. This makes it ideal for the treatment of acute and emergency situations, in which drug bioavailability should be immediate [56,57].

Independently of the intended administration route, the developed nanometric emulsions were able to increase antipsychotic drug solubility up to 4796 times, when compared to their aqueous solubility, which is quite substantial. As for the most frequently studied drugs, in general most studies involved second-generation antipsychotics, which is not surprising since they are the largest antipsychotic drug generation (when compared to first and third generations).

In what concerns the excipients selected to be part of the formulation’s composition, they were mostly chosen according to drug solubility, but also according to potential safety. Although specific safety studies were not frequently performed, it was reasonably common for authors to choose “generally recognized as safe” (GRAS) excipients, search for existent toxicity information in advance, and/or select formulas with a minimum amount of possibly toxic excipients (such as surfactants of cosolvents). Additionally, other considerations for excipient selection included: being known permeation enhancers; being P-glycoprotein inhibitors; and/or being CYP enzymes metabolism inhibitors.

As for the most used excipients, in the lipids category Capmul^®^ MCM (medium chain mono- and diglycerides) was the most utilized, either as the oily vehicle or as a hydrophobic surfactant, due to its recognized good solvent, emulsifying and bioavailability enhancement capabilities. The most used hydrophilic surfactant was Tween^®^ 80 (polysorbate 80-polyoxyethylene 20 sorbitan monooleate), which is a very well-known O/W emulsifier that has been widely used as a stabilizer in this type of formulations. As for cosolvents, Transcutol^®^ (P or HP, depending on its purity; diethylene glycol monoethyl ether) was the most used, having penetration enhancement capability as well as being a powerful drug molecule solubilizer.

Independently of composition, in general nanometric emulsions performed superiorly than the comparative controls (drug solutions or suspensions), leading to improved brain drug targeting, which was accessed by brain drug quantification (pharmacokinetics) or observation of therapeutic-like effects (pharmacodynamics) in animal models. This is probably mainly due to these formulation’s excipients (surfactants and cosolvents), which have drug permeation enhancing capability, added to a small droplet size, which leaves a large surface area available for drug diffusion and, consequently, absorption to occur at the administration site. Moreover, the addition of a mucoadhesive polymer to these preparations (especially important in the case of intranasal delivery) led to even better results, with the increased formulation retention at the administration site leading to an increased time for drug absorption to occur and, consequently, increased bioavailability.

However, some articles lacked in vivo evaluation, which is crucial for assessing the real potential of a developed formulation, especially for diseases with a brain etiology, for which in vitro experiments are not applicable for assessing drug efficacy. However, even in the articles that performed this kind of studies, it is quite difficult to compare between different drugs. Moreover, even in studies that have used the same drug a number of factors can make the comparison biased, since each experimental procedure is made of a series of variable factors. Yet, it is still possible to try to conduct a comparative analysis, even if somehow subjective. The critical analysis for individual articles has been carried out in previous Section 3.1, Section 3.2 and Section 3.3, and the comparison between articles that formulated the same drug is carried out in the following paragraphs.

There were 4 studies that developed risperidone O/W nanoemulsions, 2 for parenteral administration [19,20] and 2 for intranasal administration [21,22]. Despite both being classified as O/W nanoemulsions, the intranasal nanoemulsion was prepared by spontaneous emulsification, which is an advantage, whereas the parenteral nanoemulsion required high pressure homogenization methods. The intranasal nanoemulsion also solubilized a higher risperidone amount (3.5 vs. 1 mg/mL), which might have been due to having an overall higher number of surfactants and cosolvents in its composition. The mean droplet size was lower for the intranasal nanoemulsion, but the PDI was lower for the parenteral one, making it more homogeneous. As for viscosity, it was adapted according to the administration route, being naturally higher for the intranasal formulation. In what concerns in vivo results, both formulations had an overall good performance, being effective in decreasing induced locomotor activity. However, despite having the same animal model (rats), since the administered doses were quite different, and the quantification methods in the pharmacokinetic study were also not the same (chromatography-mass spectrometry vs. scintigraphy), it makes it hard to conclude on which formulation performed best.

Olanzapine O/W nano- and micro-emulsions were developed in 2 studies, through spontaneous emulsification, with different composition but reaching a similar drug strength and droplet size. Despite the similar droplet size, Kumar et al. [23] classified the developed formulations as nanoemulsions, whereas Patel et al. [24] considered them to be microemulsions. As mentioned in Section 1, there is no consensus in what concerns nanometric emulsions’ droplet size classification, hence in the present review we decided to go with the classification that the authors of the included articles decided upon. The formulations developed by Patel et al. had a higher viscosity, which could lead to a higher retention at the administration site, and the non-mucoadhesive nanoemulsion also had a lower PDI, making it more homogeneous and, consequently, potentially more stable and leading to a better pharmacokinetic profile. Nevertheless, similarly to risperidone, despite having the same animal model for one of the pharmacodynamic tests (mice, locomotor activity), the administered dose was 10 times higher in one of the articles, making it hard to carry out a direct and fair comparison, aside from the general conclusion that all developed formulations were effective in having therapeutic-like effects.

Nanometric emulsions containing quetiapine, for intranasal delivery, were developed in 2 studies [25,26]. The microemulsions developed by Shah et al. [26] were prepared by spontaneous emulsification, and managed to increase quetiapine solubility when compared to water, whereas the nanoemulsion developed by Boche et al. [25] had to be prepared by high sheer methods, and the drug strength in the final formulation was not reported (hence it was not possible to compare). The mean droplet size was higher for the nanoemulsion than for the microemulsions, which is in agreement with their size definition, and the PDI values were similar, thus making them all reasonably homogeneous. The microemulsions’ viscosity was low, but although the authors state that it is a quite relevant parameter, especially in intranasal delivery (for good sprayability), the viscosity of the nanoemulsion was not measured. As for the comparison of in vivo pharmacokinetic results, despite having been administered in a lower dose, the nanoemulsion of Boche et al. resulted in a brain C_max_ higher than all the microemulsions developed by Shah et al. (rats, quantification by chromatography), which makes it seem superior. Nevertheless, aside from a different dose, the sample processing procedure and tissue collection time points were also different, and hence it might not be possible to draw a comprehensive conclusion, since a comparison free of bias would only be possible if the studies had been conducted under the exact same conditions.

Paliperidone O/W intranasal nanometric emulsions were carried out in 2 studies. Despite having similar viscosities, the microemulsions developed by Patel et al. [28] reached a higher drug strength, lower droplet size, good PDI, and were prepared by spontaneous emulsification, whereas the nanoemulsion developed by Pidaparthi et al. [29] required more complex preparation methods and did not even report PDI values. Nevertheless, all formulations reportedly had therapeutic-like effects in pharmacodynamic studies (same animal model), despite the administered dose being 10 times less in the Pidaparthi et al. case.

Nanometric emulsions containing amisulpride were developed and reported in 2 articles, and were either oral SNEDDS [32], or intranasal O/W nanoemulsions [33]. Although they are different types of preparations, and meant to be administered through different administration routes, hence being difficult to compare, in general the oral SNEDDS seemed to have better formulation characteristics, since they reached a 50 times higher drug strength, and lower droplet size and PDI values (upon dilution). Nevertheless, these oral SNEDDS were not evaluated in in vivo experiments, thus their true potential in making the drug reach the brain was left unassessed.

Finally, 3 studies developed O/W aripiprazole nanoemulsions. Although the intranasal nanoemulsions [37] had a simpler preparation method (spontaneous emulsification), and the intravenous nanoemulsions [35,36] had to use high energy procedures, the intravenous preparations had a lower droplet size and PDI, with reported drug strength (whereas the intranasal nanoemulsions did not mention drug strength). The intranasal nanoemulsions were more viscous, which is in accordance with the administration route’s requirements. Nevertheless, while the intranasal formulations were deemed effective in making aripiprazole reach the brain (animal studies), with therapeutic-like effectiveness, the intravenous preparations were not evaluated in vivo, and hence their true potential was left uncertain.

With all that being said, it is quite difficult to conclude on which nanometric emulsion performed the best, since there is a quite high number of variables to account for. Nevertheless, altogether nanometric emulsions have proven to be promising strategies to improve brain bioavailability of antipsychotic drugs and should be strongly considered as promising drug delivery systems and therapeutic agents for diseases with a brain etiology. Future works should focus on the selection of the most promising formulations to assess for possible clinical efficacy, in order to gauge these formulations true potential, so that they can eventually reach the pharmaceutical market and have a positive impact on the treatment of schizophrenia and other schizoaffective disorders.

## 5. Conclusions

Nanometric emulsions have proven to be quite effective in increasing lipophilic antipsychotic drugs’ solubility (up to 4796 times, when compared to their aqueous solubility). Moreover, these formulation’s excipients (surfactants and cosolvents) have drug permeation enhancing capability, which added to a small droplet size, that leaves a large surface area available for drug absorption to occur, has resulted in the majority of the developed formulations having an improved brain drug bioavailability and/or therapeutic-like effects in in vivo experiments (when compared to drug solutions or suspensions). Additionally, intranasal administration was the preferred administration route (when compared to oral or intravenous administration), since it offers several advantages in brain drug delivery, namely direct brain drug transport through neuronal pathways, which can result in an increased and faster therapeutic effect and reduced systemic adverse events. Hence, although the number of studies is still small, and despite the fact that it is difficult to conclude on which formulation performed the best (high number of variables to account for), nanometric emulsions have proven to be good strategies for improving brain bioavailability of antipsychotic drugs, for the treatment of the highly incident and impactful disorder that is schizophrenia, making them promising therapeutic delivery platforms.

## Figures and Tables

**Table 1:** Summary of the most relevant formulation parameters of the nanometric emulsions developed in the works included in this review.

Antipsychotic Generation	Drug	Administration Route	Formulation	Preparation Method	Droplet Size (nm)	PDI	Zeta Potential (mV)	Viscosity (cP)	pH	Osmolarity/Osmolality	Ref.
First	Chlorpromazine	Oral	SNEDDS	Spontaneous emulsification	159 to 186	0.27 to 0.33	−14.1 to −21.4	NR	7.0 to 7.5	NR	[17]
	Haloperidol	Intranasal	O/W nanoemulsion	Spontaneous emulsification	210	0.40	−23.85	NR	NR	NR	[18]
Second	Risperidone	Parenteral	O/W nanoemulsions	High-pressure homogenization	160	0.10 to 0.13	−50	6 to 11	8.2 to 8.4	NR	[19,20]
		Intranasal	O/W nanoemulsions	Spontaneous emulsification	14 to 18	0.12 to 0.17	−9 to −12	225 to 250	4.5 to 5.3	270 mOsmol/L	[21,22]
	Olanzapine	Intranasal	O/W nanoemulsions	Spontaneous emulsification	20 to 24	0.25 to 0.30	−4 to −11	0.10 to 0.13	4.5 to 5.7	NR	[23]
		Intranasal	O/W microemulsions	Spontaneous emulsification	24 to 32	0.12 to 0.25	−35.14 to −42.15	75 to 93	5.9 to 6.0	NR	[24]
	Quetiapine	Intranasal	O/W nanoemulsion	Ultrasonication	144	0.19	−8.13	NR	NR	NR	[25]
		Intranasal	O/W microemulsions	Spontaneous emulsification	30 to 47	0.22 to 0.25	2.77 to 20.29	17.5 to 38.5	5.5 to 6.5	NR	[26]
	Ziprasidone	Intranasal	O/W nanoemulsion	Spontaneous emulsification	145	0.19	−30.2	183	6.5	310 mOsmol/L	[27]
	Paliperidone	Intranasal	O/W microemulsions	Spontaneous emulsification	20 to 27	0.05 to 0.12	−36.59 to −38.65	77 to 96	5.9 to 6.0	NR	[28]
		Intranasal	O/W nanoemulsion	Aqueous titration + high-pressure homogenization	38	NR	−0.009	94.5	6.5	NR	[29]
	Iloperidone	Oral	O/W nanoemulsion	Hot homogenization + ultrasonication	222	0.20	−28.9	NR	NR	NR	[30]
	Asenapine	Intranasal	O/W nanoemulsions	Aqueous titration	21	0.36	−14.1	91.7 to 235.7	NR	NR	[31]
	Amisulpride	Oral	SNEDDS	Spontaneous emulsification	16 to 22	0.09 to 0.19	NR	NR	NR	NR	[32]
		Intranasal	O/W nanoemulsions	Aqueous titration	92 to 106	0.46 to 0.51	18.22 to −16.01	8.7 to 12.6	NR	NR	[33]
	Lurasidone	Intranasal	O/W nanoemulsions	High-pressure homogenization	48	0.31	−0.19 to−0.41	119.5 to 892.3	5 to 6	NR	[34]
Third	Aripiprazole	Parenteral	O/W nanoemulsion	High-shear and high-pressure homogenization	60	0.18	−31.6	3.7	7.4	297 mOsmol/kg	[35,36]
		Intranasal	O/W nanoemulsions	Spontaneous emulsification	120 to 140	0.25 to 0.40	−16 to −19	89.3	NR	NR	[37]

NR—not reported; O/W—oil-in-water; Ref.—reference; SNEDDS—self-nanoemulsifying drug delivery system.

**Table 2:** Detailed composition of chlorpromazine and haloperidol nanometric emulsions. Excipient and drug quantities and units are shown as reported in the respective articles (*w*/*w*% for excipients, and *w*/*w*% or mg/mL for drugs). For the excipients the brand name was used (when available).

Composition		Oral Chlorpromazine SNEDDS [17] (% *w*/*w*)			Intranasal Haloperidol O/W Nanoemulsions [18]	
		With Short-Chain Triglyceride	With Medium-Chain Triglyceride	With Long-Chain Triglyceride	Non-Mucoadhesive	Mucoadhesive
Oil	Triacetin	40%	-	-	-	-
	Captex^®^ 355 ^1^	-	35%	-	-	-
	Olive oil + linseed oil	-	-	55%	-	-
	Capmul^®^ MCM ^2^	-	-	-	NR	NR
Hydrophobic surfactant	Span^®^ 20 ^3^	-	-	-	NR	NR
Hydrophilic surfactant	Tween^®^ 85 ^4^	48%	50%	40%	-	-
	Tween^®^ 80 ^5^	-	-	-	NR	NR
Cosolvent	Ethanol	5%	5%	3%	-	-
	Glycerol	5%	8%	-	-	-
	Transcutol^®^ P ^6^	-	-	-	NR	NR
Mucoadhesive and/or viscosifying agents	Pemulen™ TR-2 ^7^	-	-	-	-	0.3% *w*/*w*
Aqueous phase	Water	-	-	-	NR	NR
Drug	Chlorpromazine	2%	2%	2%	-	-
	Haloperidol	-	-	-	8.5 mg/mL	8.5 mg/mL

NR—not reported; SNEDDS—self-nanoemulsifying drug delivery system; ^1^ glyceryl tricaprylate/tricaprate; ^2^ medium chain mono- and diglycerides; ^3^ sorbitan monolaurate; ^4^ polysorbate 85-polyoxyethylene 20 sorbitan trioleate; ^5^ polysorbate 80-polyoxyethylene 20 sorbitan monooleate; ^6^ diethylene glycol monoethyl ether; ^7^ acrylates/C10–30 alkyl acrylate crosspolymer.

**Table 3:** Detailed composition of the risperidone parenteral and intranasal nanoemulsions. Excipient and drug quantities and units are shown as reported in the respective articles (*w*/*w*% for excipients, and mg/mL for drugs). For the excipients the brand name was used (when available).

Composition		Parenteral O/W Risperidone Nanoemulsions [19,20] (% *w/w*)			Intranasal O/W Risperidone Nanoemulsions [21,22] (% *w*/*w*)	
		With Tween^®^ 80	With Kolliphor^®^ P 188	With Kolliphor^®^ HS 15	Non-Mucoadhesive	Mucoadhesive
Oil	Medium chain triglycerides	16%	16%	16%	-	-
	Soybean oil ^1^	4%	4%	4%	-	-
	Capmul^®^ MCM ^2^	-	-	-	8%	8%
Hydrophobic surfactant	Soybean lecithin ^3^	2%	2%	2%	-	-
Hydrophilic surfactant	Tween^®^ 80 ^4^	2%	-	-	29.33%	29.33%
	Kolliphor^®^ P 188 ^5^	-	2%	-	-	-
	Kolliphor^®^ HS 15 ^6^	-	-	2%	-	-
Cosolvent	Benzyl alcohol	2%	2%	2%		
	Transcutol^®^ P ^7^	-	-	-	7.33%	7.33%
	Propylene glycol	-	-	-	7.33%	7.33%
Mucoadhesive and/or viscosifying agents	Chitosan	-	-	-	-	0.50%
Other components	Butylhydroxytoluene (antioxidant)	0.05%	0.05%	0.05%	-	-
	Glycerol (to adjust tonicity)	2.25%	2.25%	2.25%	-	-
Aqueous phase	Water	-	-	-	48%	48%
	Water + sodium oleate 0.03% *w*/*w* (pH 9)	qs	qs	qs	-	-
Drug	Risperidone	1 mg/mL	1 mg/mL	1 mg/mL	3.5 mg/mL	3.5 mg/mL

Qs—*quantum satis*; ^1^ Lipoid Purified Soybean Oil 700; ^2^ medium chain mono- and diglycerides; ^3^ Lipoid S 75; ^4^ polysorbate 80-polyoxyethylene 20 sorbitan monooleate; ^5^ poloxamer 188; ^6^ macrogol 15 hydroxystearate; ^7^ diethylene glycol monoethyl ether.

**Table 4:** Detailed composition of the intranasal olanzapine nano and microemulsions. Excipient and drug quantities and units are shown as reported in the respective articles (*w*/*w*% for excipients, and mg/mL for drugs). For the excipients the brand name was used (when available).

Composition		Intranasal Olanzapine O/W Nanoemulsions [23] (% *w*/*w*)		Intranasal Olanzapine O/W Microemulsions [24] (% *w*/*w*)	
		Non-Mucoadhesive	Mucoadhesive	Non-Mucoadhesive	Mucoadhesive
Oil	Capmul^®^ MCM ^1^	15%	15%	-	-
	Oleic acid	-	-	4%	4%
Hydrophilic surfactant	Tween^®^ 80 ^2^	35%	35%	-	-
	Labrasol^® 3^	-	-	12%	12%
	Kolliphor^®^ RH40 ^4^	-	-	12%	12%
Cosolvent	Ethanol	8.75%	8.75%	-	-
	Polyethylene glycol 400	8.75%	8.75%	-	-
	Transcutol^®^ P ^5^	-	-	8%	8%
Mucoadhesive and/or viscosifying agents	Chitosan ^6^	-	0.50%	-	-
	Polycarbophil AA-1	-	-	-	0.5%
Aqueous phase	Water	32.5%	32.5%	64%	64%
Drug	Olanzapine	8.5 mg/mL	8.5 mg/mL	8 mg/mL	8 mg/mL

^1^ medium chain mono- and diglycerides; ^2^ polysorbate 80-polyoxyethylene 20 sorbitan monooleate; ^3^ polyethylene glycol-8 caprylic/capric glycerides; ^4^ polyoxyl 40 hydrogenated castor oil; ^5^ diethylene glycol monoethyl ether; ^6^ medium viscosity, molecular weight 400 kDa, degree of deacetylation 83–85%.

**Table 5:** Detailed composition of the intranasal quetiapine nano and microemulsions and intranasal ziprasidone nanoemulsions. Excipient and drug quantities and units are shown as reported in the respective articles (*w*/*w*% for excipients, and mg/mL for drugs). For the excipients the brand name was used (when available).

Composition		Intranasal O/W Quetiapine Nanoemulsion [25] (% *w*/*w*)	Intranasal O/W Quetiapine Microemulsions [26] (% *w*/*w*)			Intranasal Ziprasidone O/W Nanoemulsions [27] (% *w*/*w*)		
			Non-Mucoadhesive	Mucoadhesive	With Cyclodextrins	Non-Mucoadhesive	Mucoadhesive 1 (with Chitosan)	Mucoadhesive 2 (with Carbopol)
Oil	Capmul^®^ MCM ^1^	12%	6%	6%	6%	15%	15%	15%
Hydrophilic surfactant	Tween^®^ 80 ^2^	34%	16.5%	16.5%	16.5%	-	-	-
	Labrasol^® 3^	-	16.5%	16.5%	16.5%	34.66%	34.66%	34.66%
Cosolvent	Transcutol^®^ P or HP ^4^	17%	11%	11%	11%	17.33%	17.33%	17.33%
	Propylene glycol	4%	-	-	-	-	-	-
Mucoadhesive and/or viscosifying agents	Chitosan	-	-	0.5% ^5^	-	-	0.5%	-
	Carbopol^®^ 934 ^6^	-	-	-	-	-	-	0.5%
Other components	Trimethyl-beta-cyclodextrins	-	-	-	3%	-	-	-
Aqueous phase	Water	33%	50%	50%	50%	-	-	-
	Phosphate buffer (pH 8)	-	-	-	-	33%	33%	33%
Drug	Quetiapine	NR	6 mg/mL	6 mg/mL	6 mg/mL	-	-	-
	Ziprasidone (hydrochloride)	-	-	-	-	20 mg/mL	20 mg/mL	20 mg/mL

NR—not reported; ^1^ medium chain mono- and diglycerides; ^2^ polysorbate 80-polyoxyethylene 20 sorbitan monooleate; ^3^ polyethylene glycol-8 caprylic/capric glycerides; ^4^ diethylene glycol monoethyl ether; ^5^ low molecular weight, degree of deacetylation 75–85%; ^6^ carbomer 934.

**Table 6:** Detailed composition of the intranasal paliperidone nano and microemulsions, oral iloperidone nanoemulsion and intranasal asenapine nanoemulsion. Excipient and drug quantities and units are shown as reported in the respective articles (*w*/*w*%, *v*/*v*%, *w*/*v*% or mg/mL for excipients, and mg/mL for drugs). For the excipients the brand name was used (when available).

Composition		Intranasal Paliperidone O/W Microemulsions [28] (% *w*/*w*)		Intranasal Paliperidone O/W Nanoemulsion [29] (% *w*/*w*)	Oral Iloperidone O/W Nanoemulsion [30] (mg/mL)	Intranasal Asenapine O/W Nanoemulsions [31] (% *v*/*v*)	
		Non-Mucoadhesive	Mucoadhesive			Non-Mucoadhesive	Mucoadhesive
Oil	Oleic acid	4%	4%	-	2.5	-	-
	Labrafil^®^ M 1944 CS ^1^	-	-	14.95%	-	-	-
	Soybean oil ^2^	-	-	-	200	-	-
	Capmul^®^ PG-8 ^3^	-	-	-	-	15%	15%
Hydrophobic surfactant	Egg lecithin ^4^	-	-	-	40	-	-
	Cholesterol	-	-	-	3	-	-
Hydrophilic surfactant	Tween^®^ 80 ^5^	-	-	41.14%	-	-	-
	Labrasol^® 6^	11.25%	11.25%	-	-	-	-
	Kolliphor^®^ RH40 ^7^	11.25%	11.25%	-	-	26.25%	26.25%
Cosolvent	Transcutol^®^ P or HP ^8^	7.5%	7.5%	13.71%	-	8.75%	8.75%
	Glycerol	-	-	-	22.5	-	-
Mucoadhesive and/or viscosifying agents	Polycarbophil AA-1	-	0.5%	-	-	-	-
	Carbopol^®^ 971 ^9^	-	-	-	-	-	0.5% *w*/*v*
Other components	Benzalkonium chloride (preservative)	-	-	0.02% *w*/*v*	-	-	-
Aqueous phase	Water	66%	66%	-	qs	qs	qs
	Phosphate buffer pH 6.4	-	-	qs	-	-	-
Drug	Paliperidone	5 mg/mL	5 mg/mL	2.5 mg/mL	-	-	-
	Iloperidone	-	-	-	0.4	-	-
	Asenapine (maleate)	-	-	-	-	NR	NR

NR—not reported; qs—*quantum satis*; ^1^ oleoyl polyoxyl-6 glyceride; ^2^ Lipoid; ^3^ propylene glycol monocaprylate; ^4^ egg phosphatidyl choline, EPC80; ^5^ polysorbate 80-polyoxyethylene 20 sorbitan monooleate; ^6^ polyethylene glycol-8 caprylic/capric glycerides; ^7^ polyoxyl 40 hydrogenated castor oil; ^8^ diethylene glycol monoethyl ether; ^9^ carbomer 971.

**Table 7:** Detailed composition of the amisulpride oral SNEDDS and intranasal nanoemulsions, and the lurasidone intranasal nano and microemulsions. Excipient and drug quantities and units are shown as reported in the respective articles (*w*/*w*, *w*/*v*% or *v*/*v* for excipients, and mg/g or mg/mL for drugs). For the excipients the brand name was used (when available).

Composition		Oral Amisulpride SNEDDS [32] (% *w*/*w*)			Intranasal Amisulpride Nanoemulsions [33] (% *v*/*v*)		Intranasal Lurasidone O/W Nano and Microemulsions [34] (% *w*/*w*)		
		1	2	3	Non-Mucoadhesive	Mucoadhesive Gel	Non-Mucoadhesive Nanoemulsion	Mucoadhesive Nanoemulsion	Non-Mucoadhesive Microemulsion
Oil	Capryol^TM^ 90 ^1^	10%	20%	30%	-	-	-	-	-
	Maisine^®^ CC ^2^	-	-	-	4%	4%	-	-	-
	Capmul^®^ MCM EP ^3^	-	-	-	-	-	10%	10%	10%
	Capmul^®^ MCM C8 EP ^4^	-	-	-	-	-	10%	10%	-
Hydrophilic surfactant	Cremophor^®^ RH40 ^5^	30%	30%	40%	-	-	-	-	*
	Cremophor^®^ EL ^6^	-	-	-	-	-	25%	25%	-
	Labrasol^® 7^	-	-	-	33.3%	33.3%	-	-	*
Cosolvent	Transcutol^®^ P or HP ^8^	60%	50%	30%	16.7%	16.7%	-	-	*
Mucoadhesive and/or viscosifying agents	Poloxamer 407	-	-	-	-	7.82% *w*/*v*	-	-	-
	Gellan gum	-	-	-	-	0.138% *w*/*v*	-	-	-
	Polycarbophil AA-1	-	-	-	-	-	-	0.5%	-
Aqueous phase	Water	-	-	-	46%	46%	55%	55%	50%
Drug	Amisulpride	50 mg/g	50 mg/g	50 mg/g	1 mg/mL	1 mg/mL	-	-	-
	Lurasidone (hydrochloride)	-	-	-	-	-	10 mg/mL	10 mg/mL	10 mg/mL

* Labrasol + Cremophor + Transcutol = 40% *w*/*w*; SNEDDS—self-nanoemulsifying drug delivery system; ^1^ propylene glycol monocaprylate (type II); ^2^ glyceryl monolinoleate; ^3^ medium chain mono- and diglycerides; ^4^ glyceryl monocaprylate; ^5^ polyoxyl 40 hydrogenated castor oil; ^6^ polyoxyl 35 castor oil; ^7^ polyethylene glycol-8 caprylic/capric glycerides; ^8^ diethylene glycol monoethyl ether.

**Table 8:** Detailed composition of the intranasal and intravenous aripiprazole nanoemulsions. Excipient and drug quantities and units are shown as reported in the respective articles (*w*/*w*%). For the excipients the brand name was used (when available).

Composition		Intravenous Aripiprazole O/W Nanoemulsion [35,36] (% *w*/*w*)	Intranasal Aripiprazole O/W Nanoemulsions [37] *	
			Non-Mucoadhesive	Mucoadhesive
Oil	Palm kernel oil esters	3.00%	-	-
	Capmul^®^ PG-8 ^1^	-	20%	20%
Hydrophobic surfactant	Soybean lecithin ^2^	2.00%	-	-
Hydrophilic surfactant	TPGS ^3^	-	36.67%	36.67%
	Tween^®^ 80 ^4^	1.00%	-	-
Cosolvent	Transcutol^®^ HP ^5^	-	18.33%	18.33%
	Glycerol	2.25%	-	-
Mucoadhesive and/or viscosifying agents	Carbopol^®^ 971 ^6^	-	-	0.5% *w*/*w*
Aqueous phase	Water	91.75%	35%	35%
Drug	Aripiprazole	0.10%	NR	NR

NR—not reported; * percentual units not specified; ^1^ propylene glycol monocaprylate; ^2^ Lipoid S75; ^3^ D-α tocopheryl polyethylene glycol 1000 succinate, ester of vitamin E succinate with polyethylene glycol 1000; ^4^ polysorbate 80-polyoxyethylene 20 sorbitan monooleate; ^5^ diethylene glycol monoethyl ether; ^6^ carbomer 971.

## Data Availability

Not applicable.

## Abbreviations

AUC—area under the “drug concentration vs. time” curve; C_max_—maximum drug concentration; FDA—Food and Drug Administration; GRAS—generally recognized as safe; O/W—oil-in-water; NR—not reported; SNEDDS—self-nanoemulsifying drug delivery system; TPGS—D-α tocopheryl polyethylene glycol 1000 succinate; T_max_—time to reach maximum drug concentration.
